# Supplementary material for: ‘Respond’—A novel approach to healthcare delivery for people seeking asylum
Source: PLOS Glob Public Health. 2026 Apr 28;6(4):e0005006. doi: 10.1371/journal.pgph.0005006 (PMC13123967; doi:10.1371/journal.pgph.0005006)
Supplement: S1 Text — (DOCX) [file pgph.0005006.s001.docx]

# RESPOND / University College London Hopsital / Hospital for Tropical Diseases

# Migrant Health Screening Questionnaire – completed on routinely collected electronic health record in EPIC.

**Content:**

1. Immediate Health Needs
2. Demographics
3. Journey
4. Paediatric Questions
5. Problem list and medications / orders
6. Allergies
7. Further Screening
8. Female Specific Questions
9. Cancer Screening
10. Immunisation Questionnaire
11. Immunisation record on EPIC
12. Social History
13. Sexual Health
14. Child Safeguarding Questionnaire
15. Adult Safeguarding
16. Mental Health Questionnaire
17. RHS-15
18. Vital Signs

# Immediate Health Needs:

- Are there any signs of acute illness / immediate health concerns?

NO – proceed to introductions

YES – refer to duty doctor

- Does patient currently have any symptoms? (circle as appropriate)

*Fever Rash Diarrhoea Vomiting Cough > 2 weeks Night sweats Chest pain*

*Other (please give details):*

If:

- Fever, rash or diarrhoea and visited a tropical country in the last year, refer to Hospital for Tropical Diseases.
- Acutely unwell / any other symptoms, discuss with duty doctor.

# Demographics

- Accompanying family members (circle as appropriate): *Yes / No*
- If yes, provide name and date of birth:

*Mother Father Stepmother Stepfather Foster Parents*

*Grandmother (Maternal) Grandfather (Maternal) Grandmother (Paternal)*

*Grandfather (Paternal) Aunt Uncle Significant other*

*Spouse / Partner Other (please specific)*

- Country of Birth (Please specify)
- What is your ethnicity? (Circle as appropriate)

*White British White Irish Other White Background Mixed White & Black*

*Mixed White & Asian Other Mixed Background Asian Indian Asian Pakistani*

*Asian Bangladeshi Other Asian Background Black Caribbean Black African*

*Other Black Background Chinese Other Ethnic Group Not Stated / Unknown*

*Not Yet Asked*

- What is your religion? (please specify)
- Current type of accommodation (circle as appropriate)

*Initial accommodation Dispersal accommodation Detention Staying with friends / family*

*Other (please specify)*

- Do you have any family or other links in the UK (circle as appropriate)

*YES / NO*

If YES, please specify

# Journey

- Date of departure from country of origin:
- Reason for leaving country (circle as appropriate)

*Persecution Torture Sexual violence FGM Modern slavery*

*Conflict Famine Other, please specify*

- Date of arrival in country:
- Duration of travel:
- Mode of transport to UK (circle as appropriate)

*Formal route (Formal is where a visa is granted or available for the journey*

*Informal route*

- Number of countries transited through: (please specify)

# Paeds Questions

- All children (0 – 17 years)

Gestation at delivery (circle as appropriate)

*In weeks (if known) Premature Not premature*

Location of delivery: (please specify)

- Children age 0 – 4 years

Is your child attending a nursery OR a children’s group OR looked after by another adult?

*If YES (provide name, place etc) / NO (circle as appropriate)*

- Children age 5 – 17 years

Is your child attending school?

*If YES (provide name and address of school) / NO (circle as appropriate)*

Are there concerns about child’s progress at school?

*If YES (specify reason) / NO (circle as appropriate)*

Does your child have SEN (Special educational needs)?

*YES / NO (circle as appropriate)*

Has your child previously attended a special needs school?

*YES / NO (circle as appropriate)*

SEN with EHCP (Educational Health and Car Plans) without EHCP, unsure?

*With EHCP Without EHCP Unsure (circle as appropriate)*

# Problem list and medications / orders

- Record any medical problem in the past medical history
- Medication history

# Allergies

Allergies / Contraindications

Know Allergies (circle as appropriate)

*YES (please specify) / NO*

Mark as Reviewed / Unable to assess (circle as appropriate)

# Further Screening

- Further screening questionnaire – Do you currently or have you in the past (including childhood) had any of the following? (circle as appropriate)

*Heart disease High blood pressure High Cholesterol Asthma*

*Other lung disease/problems Stomach or bowel problems Liver Problems*

*Kidney or urinary tract problems Ear/Nose/Throat problems Diabetes*

*Thyroid disease Other hormonal or endocrine conditions Eczema*

*Psoriasis Other skin conditions Muscle/joint/back problems Epilepsy*

*Stroke Other neurological condition/problems (brain or nerve) Depression*

*Anxiety Eating Disorder Other mental health disorder*

*Cancer or other malignancy Immunosuppressed due to condition or medication*

*No spleen/poorly functioning spleen HIV positive*

*Sickle cell disease or other haemaglobinopathy Deep vein thromboses Blood disorder*

*Gynaecological problems*

- Have you been admitted to hospital or been under the care of a specialist in the last 5 years?

*YES / NO (circle as appropriate)*

If yes, please provide details

- Do you have any pending medical investigations or treatment?

*YES / No (circle as appropriate)*

If yes, please provide details

- Have you ever had surgery?

*YES / NO (circle as appropriate)*

If yes, please provide details of type and dates:

- When did you last see a dentist? (circle as appropriate)

*0-6 months 6-12 months 12-24 months 24-36 months >36 months*

- Do you use a toothbrush and toothpaste? (circle as appropriate)

*YES / NO*

- If yes, do you brush your teeth twice a day? (circle as appropriate)

*YES / NO*

- Do you have any dental plan? (circle as appropriate)

*YES / NO*

If yes – signpost to NHS 111

- Do you have any concerns about your or your child’s vision? (circle as appropriate)

*YES / NO*

- Do you wear glasses (circle as appropriate)

*YES / NO*

- Do you have any problems with you / your child’s hearing? (circle as appropriate)

*YES / NO*

- Have you / they had a hearing test? (circle as appropriate)

*YES / NO*

- Do you / they wear a hearing aid? (circle as appropriate)

*YES / NO*

# Female specific questions

- Do you have menstrual periods? (circle as appropriate)

*YES NO Prefer not to say*

Date of LMP:

- Do you have concerns about your periods? (circle as appropriate)

*YES / NO*

If yes, please circle as appropriate

*Pain Very heavy +/- flooding Irregular*

- Do you have access to menstrual hygiene products e.g. cups, tampons, pads? (circle as appropriate)

*YES / NO*

- What do you use when you menstruate? (circle as appropriate)

*Cups Tampons Pads Other – please specify*

- Have you had any information / education about menstrual hygiene? (circle as appropriate)

*YES NO Don’t know*

If patient response is no or don’t know, provide patient with information.

- Have you ever been pregnant or delivered a baby? (circle as appropriate)

*YES NO Prefer not to answer*

If yes, please provide details:

- Are you currently pregnant? (circle as appropriate)

*YES NO Don’t know*

- - If don’t know, would you like a pregnancy test? (circle as appropriate)

*YES / NO*

- - If yes, have you received antenatal care? (circle as appropriate)

*YES / NO*

- - - If yes, where have your received antenatal care? (please specify)
  - If no, are you on contraception?

*YES / NO*

- - - If yes, what type of contraception are you on? (please specify)
    - If no, would you like to start contraception? (circle as appropriate)

*YES / NO*

- Are you currently breast feeding?

*YES / NO*

# Cancer Screening

- Have you ever had a screening test for cancer? (circle as appropriate)

*YES / NO*

- If female, 26 – 49 years old: what year was your last cervical smear test (please specify)

[Hint, every 3 years]

- If female, 50 – 71 years old: what year was your last breast cancer screening test (please specify)

[Hint, every 5 years]

- If male or female, 60-74 years old: what year was your last bowel cancer screening test (please specify)

[Hint, every 3 years]

- If male, 65 years and over: what year was your last AAA screening test? (please specify)

[Hint, one off]

# Immunisation questionnaire

- Have you had previous immunisations? (please circle as appropriate)

*YES/NO*

- Have you had COVID vaccine? (please specify)

Date of first dose:

Date of second dose:

- Can you confirm you or your child’s immunisation history? (please specify)

# Social History

- Tobacco

Tobacco use (circle as appropriate)

*YES NO Previously*

*Start Date:*

*Quit Date:*

Types (circle as appropriate)

*Cigarettes Pipe Cigars E-cigarettes Shisha*

Packs per day (circle as appropriate)

*0.25 0.5 1 1.5 2 3*

Number of years you have smoked? (circle as appropriate)

*0.5 1 2 3 4 5 10 15*

Additional comments:

Smokeless Tobacco use? (circle as appropriate)

*YES / NO*

Types (circle as appropriate)

*Snuff / Chew*

Smokeless Tobacco Quit Date:

- **Alcohol**

Alcohol use? (circle as appropriate)

*YES Not Currently Never Defer*

Drinks per week (please specify)

*Glasses of wine*

*Cans of beer*

*Shots of spirits*

*Standard drinks or equivalent*

*Pint of Beer / Cider 3.6%*

*Pints of Beer / Cider 5.2%*

*Bottle of Beer / Cider (330mls, 5%)*

*Can of Beer / Cider (440 mls, 5.5%*

*Glass of wine (Standard 175mls, 12%)*

*Glass of Wine (Large 250mls, 12%)*

*Glass of Prosecco / Champagne (125mls, 12%)*

*Spirits (one shot 35mls, 40%)*

*Spirits (two shots 70mls, 40%)*

*Alcopop (275mls, 5.5%*

Alcohol consumption per week: (please detail below)

- **Substance Use**

Drug Use (circle as appropriate)

*YES Not Currently Never Defer*

Types (circle as appropriate)

*Cannabis Cocaine Hallucinogens Inhalants Methamphetamines*

*Prescribed opioids Sedatives Stimulant Street opioids*

Additional comments: (please specify)

# Sexual Health

- Have you received SRE (sex and relationships education)? (circle as appropriate)

*YES / NO*

- Are you able to answer some questions about sex and relationships? (circle as appropriate)

*YES NO Unsure*

- Are you in a relationship or seeing someone at the moment? (circle as appropriate)

*YES NO Prefer not to answer*

- Do you identify as gay, straight or other? (circle as appropriate)

*Heterosexual/straight Gay Bi Prefer not to say*

*Prefer to self-specify*

- Are you having sex / sexually active (circle as appropriate)

*YES NO Prefer not to say*

- Do you know that the legal age of consent to have sex in the UK is 16 years old? (circle as appropriate)

*YES NO Prefer not to answer*

- Do you know about safe sex? (circle as appropriate)

*YES NO Prefer not to say*

- Do you know where to go to get help and advice regarding sexual health (circle as appropriate)

*YES NO Prefer not to say*

# Child Safeguarding questionnaire

- School / Childcare Type (please specify)

School name:

School / Childcare Borough:

- Do you have a social worker (circle as appropriate)

*YES NO Other – please specify*

Details of social worker:

- Demographics completed including contact details and GP (circle as appropriate)

*YES / NO*

- Safe in Home (circle as appropriate)

*YES NO UTA – unable to assess Other – please specify*

- Safe in Relationship (circle as appropriate)

*YES NO UTA – Unable to assess Other – please specify*

- Have there been threats or direct abuse of you or your children? (circle as appropriate)

*YES NO UTA – Unable to assess Other – please specify*

- When did the abuse occur? (circle as appropriate)

*< 1 week < 1 month 1 – 6 months ago 6 – 12 months ago > 1 year*

- Do you feel you are still at risk? (circle as appropriate)

*YES NO UTA – Unable to access Other – please specify*

- Have you ever had Female Genital Mutilation (FGM, ‘Cutting’ or genital piercing performed? (Circle as appropriate)

*YES / NO*

- - Client’s report of her FGM type (circle as appropriate)

*Type 1 Type 2 Type 3 Type 4*

*Does not know type – please comment*

*Girl / Woman did not know or would not say – please comment*

- - Age Category at FGM (circle as appropriate)

*Under 1year 1 and under 5 years 5 and under 10 years*

*10 and under 15 years 15 and under 18 years 18 years and older*

*Girl / Woman did not, or would not say – please comment*

- - Country of origin: (if different from country of birth)
  - Country FGM took place:
  - Number of daughters under 18 years of age:
  - Number of sisters under 18 years of age:
  - Female relatives affected by FGM (circle as appropriate)

*None Do not know Would not say Mother Daughter(s)*

*Elder sister(s) Younger sister Cousin(s) Aunt(s)*

*Niece(s) Grandmother Other – please specify*

- Consent obtained for information sharing and School Nurse / Health Visitor and Social Services? (circle as appropriate)

*YES / NO*

- Any other safeguarding concerns? (circle as appropriate)

*YES / NO*

- Brief summary of area of concern and action taken:
- Safeguarding Referral completed? (circle as appropriate)

*YES / NO*

- Type of Referral? (circle as appropriate)

*Health Visitor School Nurse Safeguarding Meeting (ED Patients Only)*

- Reason for referral? (Please specify)
- Referral to FGM Specialist Service
  - Accepted offer to referral to FGM specialist service? (circle as appropriate)

*YES / NO*

- Reason(s) for Declining Referral to FGM specialist service? (circle as appropriate)

*Previous vaginal delivery Has enough information about FGM*

*Previously seen at current / other hospital No issue relating to FGM*

*No reason given*

- Was information given regarding FGM? (circle as appropriate)

*YES / NO*

- Harmful effects of FGM – advised on health implication of FGM (circle as appropriate)

*YES / NO*

- FGM Law in the UK – advised that FGM is illegal in the UK (circle as appropriate)

*YES / NO*

- NHS Data Collection - Informed of NHS data collection regarding FGM (circle as appropriate)

*YES / NO*

- Was FGM Healthcare Passport Given? (circle as appropriate)

*YES / NO*

- Information Sharing System for New-born Female Infants of Mothers with FGM – information sharing discussed (circle as appropriate)

*YES / NO*

# Adult Safeguarding questionnaire

- Is anyone causing you harm in your life? (circle as appropriate)

*YES / NO*

- Do you feel safe? (circle as appropriate)

*YES / NO*

- Is anyone controlling you or what you do with your money? (circle as appropriate)

*YES / NO*

- Has anyone ever hurt you physically or forced you to do something sexually you don’t want to do? (circle as appropriate)

*YES / NO*

- Is anyone being violent towards you? (circle as appropriate)

*YES / NO*

- Some people in your position have experienced torture / ill treatment – has that ever happened to you? (circle as appropriate)

*YES / NO*

- Did anything bad happen to you during your journey? (circle as appropriate)

*YES / NO*

- Has anyone forced you to leave your country / make this journey? (circle as appropriate)

*YES / NO*

- I know that FGM is common where you are from – has that ever happened to you? (circle as appropriate)

*YES / NO*

If yes, complete the relevant FGM questionnaire

- Is this person at risk of radicalisation? (circle as appropriate)

*YES / NO*

**Questions for the healthcare workers completing the questionnaire**

- Is this person known to have a learning disability? (circle as appropriate)

*YES / NO*

- Do you think this person would benefit from input from the learning difficulties team? (circle as appropriate)

*YES / NO*

- Consent obtained for information sharing with other healthcare workers at a multidisciplinary meeting? (circle as appropriate)

*YES / NO*

- Consent obtained for information sharing with social services (circle as appropriate)

*YES / NO*

**Note: Clinicians should aim to gain consent to share information but should be mindful of situations where to do so would place a child or adult at increased risk of harm. Information may be shared without consent if a practitioner has a reason to believe that there is good reason to do so, and that the sharing of information will enhance the safeguarding of a child or adult in a timely manner. When decisions are made to share or withhold information, practitioners should record who has been given the information and why.**

Safeguarding Concern (circle as appropriate)

*YES / NO*

If YES, state which category:

# Mental health questionnaire

- Are you currently suffering from any mental health problems? (circle as appropriate)

*YES / NO*

- Have you previously seen or are you seeing a mental health professional? (circle as appropriate)

*YES / NO*

- Have you thought about hurting yourself? (circle as appropriate)

*YES / NO*

- Have you thought about hurting others? (circle as appropriate)

*YES / NO*

- Do you have any problems sleeping? (circle as appropriate)

*YES / NO*

If YES, please comment:

# RHS – 13 Refugee Health Screener

Symptoms

1. Muscle, bone, joint, pains (circle as appropriate)

0-Not at all 1-A little 2-Moderately 3-Quite a bit 4-Extremely

1. Feeling down, sad or blue most of the time (circle as appropriate)

0-Not at all 1-A little 2-Moderately 3-Quite a bit 4-Extremely

1. Too much thinking or too many thoughts (circle as appropriate)

0-Not at all 1-A little 2-Moderately 3-Quite a bit 4-Extremely

1. Feeling helpless (circle as appropriate)

0-Not at all 1-A little 2-Moderately 3-Quite a bit 4-Extremely

1. Suddenly scared for no reason (circle as appropriate)

0-Not at all 1-A little 2-Moderately 3-Quite a bit 4-Extremely

1. Faintness, dizziness or weakness (circle as appropriate)

0-Not at all 1-A little 2-Moderately 3-Quite a bit 4-Extremely

1. Nervousness of shakiness inside (circle as appropriate)

0-Not at all 1-A little 2-Moderately 3-Quite a bit 4-Extremely

1. Feeling restless, can’t sit still (circle as appropriate)

0-Not at all 1-A little 2-Moderately 3-Quite a bit 4-Extremely

1. Crying easily (circle as appropriate)

0-Not at all 1-A little 2-Moderately 3-Quite a bit 4-Extremely

1. Had the experience of reliving the trauma; acting or feeling as if it were happening again? (circle as appropriate)

0-Not at all 1-A little 2-Moderately 3-Quite a bit 4-Extremely

**The following symptoms may be related to traumatic experiences during war and migration. How much in the past month have you:**

1. Been having PHYSICAL reactions (for example, break out in sweat, heart beats fast) when reminded of the trauma? (circle as appropriate)

0-Not at all 1-A little 2-Moderately 3-Quite a bit 4-Extremely

1. Felt emotionally numb (for example, feel sad but can’t cry, unable to have loving feelings)? (circle as appropriate)

0-Not at all 1-A little 2-Moderately 3-Quite a bit 4-Extremely

1. Been jumpier, more easily startled (for example, when someone walks up behind you)? (circle as appropriate)

0-Not at all 1-A little 2-Moderately 3-Quite a bit 4-Extremely

# Vital Signs

Taken on

Date:

Time:

BP:

Site:

Position:

Pulse:

Temp:

Source:

Weight:

Height:

HC:

Resp:

Sp02:
