# Supplementary material for: ‘Respond’—A novel approach to healthcare delivery for people seeking asylum
Source: PLOS Glob Public Health. 2026 Apr 28;6(4):e0005006. doi: 10.1371/journal.pgph.0005006 (PMC13123967; doi:10.1371/journal.pgph.0005006)
Supplement: S2 Text — (DOCX) [file pgph.0005006.s002.docx]

**sService Evaluation of Respond – Qualitative arm**

**Aim:** To understand the perspectives of service users and providers of the Respond service

**Objectives:**

1. To gain the perspective of service users and providers regarding their experience of the Respond service
2. To ensure the Respond service is responsive to the needs of the intended population

**Method:**

We will be conducting 1:1 interviews with asylum-seekers (AS) who have recently undergone Respond health screening, service providers and other key stakeholders to explore their experience of and attitudes towards the Respond service.

*Sample:* Purposive sampling of service users who have undergone Respond screening. To reduce any negative impact on mental health and apply a trauma informed approach, we will only contact those with a Refugee Health Screener-13 (RHS-13) score of 20 or less. We will invite key providers who have liaised with Respond to garner their perspectives including GPs, IIHPs, commissioners, paediatricians, safeguarding specialists, mental health specialists, health visitors and family support workers.

*Interviews:* Semi-structured interviews will be conducted in person where possible with a translator as required. An interview guide structure will be followed with room to explore topics further. Interviews will last approximately 20minutes.

*Analysis:* A thematic analysis using Braun & Clarkes (2006) six point guide will be used to analyse data. This will involve familiarisation of the data, deciding on codes followed by themes. Three individuals will conduct the analysis on all interview data in order to reduce bias.

**Semi-structured interview – key providers**

- Where do you work? And in what role? In what capacity do you encounter AS?
- How have you come across Respond in your day-to-day work? – in what ways? How often? Where?
- What is your experience of the Respond service? Positive and negative experiences
- How has Respond affected your work?
- What is your perception of the impact of the Respond service on the asylum-seeking people you work with?
  - How do you feel Respond has changed providing health services for asylum-seeking people?
  - Can you give an idea about what it was like before Respond existed?
- Do you have any improvement suggestions?

| **Semi-structured interview – service users** | | |
| --- | --- | --- |
| Key area | Themes | Questions |
| Introduction | Study aim  Why invited to participate  Confirm consent including to be recorded & any questions | **Remember to enable Record Function on Teams during this section**  Respond, the clinic that you were assessed in, is a new service specifically for asylum-seekers in the NHS. We would like to gather some feedback from people seen in the service to make sure Respond is reaching its aim of improving care for asylum seekers. Your responses are anonymous and will not affect your clinical care in any way.  The interview will be recorded until we have analysed the responses, after which it will be deleted.  Do you have any questions? Do I have your consent to continue? |
| Participant background | Getting to know each other + building rapport | You were seen in the Respond service on xx date… |
| Access to care – dimension 1 | Approachability – ability to perceive | Can you describe your experience of your Respond screening appointment?  Did you know what the appointment was about before you arrived?  Did you receive any information about how to get to your appointment?  How did you receive this information?   - E.g. (phone/text/MyCare)   Did you understand the information provided before your appointment?   - Why/Why not? |
| Access to care – dimension 2 | Acceptability – ability to seek | Was the content of the appointment relevant to you?   - - How so?   Did the appointment cover all the concerns you wanted to talk to a health care professional about?   - - If no, why not?   Was there enough time in the appointment?   - - If no, why not?   We cover lots of different areas of health in the Respond clinic, did you feel this was too much to cover in one appointment?   - If no, why not? - If yes, in what way?   Did you feel the healthcare professional that assessed you was adequately trained to meet your needs? |
| Access to care – dimension 3 | Availability and accommodation – ability to reach | Where was your appointment?  Did you like having the appointment in xx location?   - - Why/why not?   Would you prefer to have this in xx location? Eg GP/hospital/community  Was the time of the appointment convenient to you?   - - Why/why not? |
| Thanks and close | Anything else to add  Any further questions  Thanks and information about how to receive study results if interested | Would you recommend this service to your friends or family?   - - Why/why not?   What would make this better/what could we do differently  Any further suggested improvements to care? |
