# Supplementary material for: ‘Respond’—A novel approach to healthcare delivery for people seeking asylum
Source: PLOS Glob Public Health. 2026 Apr 28;6(4):e0005006. doi: 10.1371/journal.pgph.0005006 (PMC13123967; doi:10.1371/journal.pgph.0005006)
Supplement: Attachment — Submitted filename: Response to reviewers.docx [file pgph.0005006.s003.docx]

Dear Editor and Reviewers,

We sincerely thank you for your thoughtful and constructive comments on our manuscript. We have carefully considered each point raised and have revised the manuscript accordingly. Below, you can find the details of our response to each one of the points raised, with the indication of the changes made in the revised version. Line numbers refer to the updated manuscript.

**Response to reviewers**

**Reviewer #1**

**Reviewer comment:**

*Clarity of study design and objectives (Lines 42–47, 118–125, 146–166)
Please make the objectives clearer. At present, it is not obvious whether the study is primarily descriptive (health needs), an assessment of feasibility, or an evaluation of service impact. Kindly edit the introduction and methods to set out primary and secondary aims more explicitly. It may also help to align the reporting with STROBE (quantitative) and COREQ (qualitative).*

**Reply:**

Thank you for your comment. We have now specified study design in lines 79-84.

We have incorporated both reviewers’ comments and clarified that the aim of the study is to describe the experience of the Respond pilot and clarified the main objectives for the quantitative data analysis and the semi-structured interviews.

Reporting has been aligned with STROBE and COREQ guidelines whenever possible, with the limitation of the interviews being conducted under the framework of an NHS service evaluation and not qualitative research.

----

**Reviewer comment:**

*Ethical approval timing (Lines 167–188)
There appears to be a mismatch between the timing of ethics approval (August 2024) and the interview period (May–July 2022). Please clarify how interviews were governed ethically, including how consent, confidentiality and safeguarding were ensured. Kindly check if this information is already in the methods and expand if needed.*

**Reply:**

Thank you for your comment. To provide more clarity, ethics approval followed two separate processes for the quantitative data analysis and the semi-structured interviews.

Ethical approval for the data analysis was approved by the Health Research Authority, details of approval number can be found in line 177-178.

The semi-structured interviews were conducted as a service evaluation project at University College London Hospital. As per HRA guidelines (<https://www.hra.nhs.uk/approvals-amendments/what-approvals-do-i-need/>), a service evaluation in the NHS would not require Health Research Authority approval but internal approval only. This was sought following established internal pathways and approved by the Division of Infection Governance Committee in May 2022. As such, the project adhered to the committee's guidelines, which included assessing the potential impact on participants, ensuring data privacy, and obtaining appropriate consent where required. The governance process of the interviews is described in the methods section (line 160-165).

---

**Reviewer comment:**

*Sampling and selection bias (Lines 189–200; 300–309)
For the quantitative analysis: Kindly add a simple flow diagram showing the pathway from eligibility to attendance and inclusion. This would make the sample derivation transparent.
For the qualitative analysis: Restricting to English-speaking, digitally literate participants with low RHS-13 scores may bias the sample. Please acknowledge this limitation more clearly in the text.*

**Reply:**

Thank you for your suggestions. A flow diagram has been added to show the pathway to inclusion. This has been inserted as figure 2 in the manuscript (line 200) and uploaded as a separate file. We have discussed the bias of the sample for the interviews in the ‘Limitations’ section lines 578-583.

---

**Reviewer comment:**

*Measurement issues (Lines 240–279)
Mental health: Please state exactly which tool was used for routine assessment, the cut-offs applied and whether interpreter support was available.
Women’s health: Kindly correct the denominator for cervical screening, since in the UK the eligible age is 25–64 rather than 15–49.
Vitamin D: Please convert results from ng/mL to nmol/L and use standard cut-offs.
HbA1c: Kindly report in mmol/mol rather than “mmol.”
Stool testing: The switch from microscopy to Novodiag (Line 143) introduces heterogeneity. Please highlight this in the limitations.*

**Reply:**

Thank you for your comments on measurements issues. We have addressed them as below:

- Mental health: in lines 295-301, we have clarified that the questions used to assess mental health are those included in our health assessment questionnaire available in the appendix. We have clarified that the tool used to assess mental health was the Refugee Health Screener 13, which is a validated in assessing psychological distress in the refugee population. Reference have been provided in the text. Interpreters were used in consultations when needed. Please see line 198.
- Women’s health: many thanks for pointing this out. The denominator has now been changed to 25-64 and proportions recalculated using this denominator.
- Vitamin D:

We have changed this. Apologies this was a mistake in reporting the measurement unit. The cutoff used are derived from national guidelines. <https://cks.nice.org.uk/topics/vitamin-d-deficiency-in-adults/>

- HbA1c:

This has been changed. It was a mistake in reporting the measurement unit.

- Stool testing:

We acknowledge that the switch from microscopy to Novodiag may introduce heterogeneity. However, we believe that detailed analysis and discussing the limitations of this bias is beyond the scope of this publications.

---

**Reviewer comment:**

Statistical analysis (Lines 146–167; 205–239)
The results are entirely descriptive. Kindly add confidence intervals for key proportions.

**Reply:**

Many thanks for the comment. I have now added confidence intervals to the tables and the results session.

---

**Reviewer comment:**

*Linkage to care and outcomes (Lines 244–299)
The discussion suggests improved linkage to care, but no data are shown.*

**Reply:**

Thank you for your comment. Data on improved linkage to care are yet to be published. To acknowledge that this improvement is a possibility rather than a certainty, we have changed the wording in line 518-520

---

**Reviewer comment:**

*Qualitative analysis transparency (Lines 168–188; 300–336; 340–397)
Kindly expand the description of how coding and theme development were conducted, whether saturation was reached and how disagreements were resolved. Please also consider adding a table that links themes with quotes for clarity.*

**Reply:**

Thank you for your comment. The interviews were part of a service evaluation rather than a qualitative research study. Therefore, we used a qualitative methodology to ensure meaningful data analysis when relevant, given the qualitative nature of the data, but it was not possible to apply the same level of rigour. We acknowledge this in the limitations (line 578-580)

----

**Minor suggestions comment:**

1. The abstract would benefit from being restructured in the standard PLOS format, with clear sections for Background, Methods, Findings and Interpretation.
2. Throughout the manuscript there are several instances of “Error! Reference source not found.”
3. Terminology is used inconsistently across the paper—different acronyms such as PSA, PSAR, CYPSAR-U and CYPSAR-A appear.
4. Please also standardise the units to UK conventions: mmol/mol for HbA1c, nmol/L for vitamin D and mmHg for blood pressure. In the women’s health section, the phrase “living with HBV” should be clarified as “surface antigen positive,” and it would be helpful to note the follow-up process.

Reply:

Thank you for all your minor suggestions. We have provided a reply as below.

1. We are unable to make this change as PLOS Global Public Health as a different abstract format without sections headings.
2. We have cross-checked references in our copy and we do not seem to encounter in the same message.
3. Thank you. We have checked and removed PSAR for consistency. We kept the remainder of the acronyms mentioned as they describe different populations and are therefore important to differentiate.
4. We have standardised the units to UK conventions. Regarding the use of the term people living with HBV, we decided to use the term as per people first terminology. This is recommended to reduce stigma and discrimination. We have added a note at the bottom of the table to clarify. Describing the follow-up process is out of the remit of this paper. However, we clarify that all infections have been referred to follow up in line 277.

**Reviewer # 2**

**Reviewer 2 comment:**

I read Respond's evaluation very carefully. The article describes the importance of participation in assessing the components involved in healthcare delivery for people seeking asylum.
I draw on what was stated in the Limitations section: "findings cannot be generalized to PSA populations elsewhere, within whom demographics may also vary geographically." I comment that it is inconsistent to call it an evaluation model, and even worse, a care new model, because a model is precisely something to be generalized.
I suggest changing the name "model" to "experience" because, additionally, sharing a good, comprehensive, or holistic experience seems like an invitation to observe and even adapt it to other conditions and realities, which would be the goal of this article.

**Reply:**

Many thanks for your comment. We have now clarified explicitly in the introduction, aims and methods and modified the term to experience. We have also ensured that this is borne in mind elsewhere when discussing potential value of the experience in informing future work.

**Editor’s comments**

1.  Please send a completed 'Competing Interests' statement, including any COIs declared by your co-authors. If you have no competing interests to declare, please state "The authors have declared that no competing interests exist". Otherwise please declare all competing interests beginning with the statement "I have read the journal's policy and the authors of this manuscript have the following competing interests:"

**Reply:**

We confirm that the authors have declared that no competing interests exist.

2. In the online submission form, you indicated that Due to the sensitive nature of the data and the specific vulnerability of the asylum-seeking population, public sharing of the dataset is restricted to avoid potential participant identification. However, de-identified data supporting the findings of this study are available from the corresponding author upon reasonable request.

All PLOS journals now require all data underlying the findings described in their manuscript to be freely available to other researchers, either 
1. In a public repository, 
2. Within the manuscript itself, or 
3. Uploaded as supplementary information.

This policy applies to all data except where public deposition would breach compliance with the protocol approved by your research ethics board. If your data cannot be made publicly available for ethical or legal reasons (e.g., public availability would compromise patient privacy), please explain your reasons by return email and your exemption request will be escalated to the editor for approval. Your exemption request will be handled independently and will not hold up the peer review process, but will need to be resolved should your manuscript be accepted for publication. One of the Editorial team will then be in touch if there are any issues.

**Reply:**

While we recognise the importance of data sharing, we are concerned about the sensitive nature of the data. Asylum seekers are a very vulnerable population and the implications of identification are very significant, particularly given political sensitivities. Even when anonymised, there may be a risk of identification where characteristics are relatively rare (e.g. <5 individuals in the cohort). We would be grateful if this exemption request could be escalated to the editor. We would be open to discuss further.

3. We do not publish any copyright or trademark symbols that usually accompany proprietary names, eg (R), (C), or TM  (e.g. next to drug or reagent names). Please remove all instances of trademark/copyright symbols throughout the text, including ©, ™ on page 7, 8, 9.

**Reply:**

We have removed symbols from the text.

4. Some material included in your submission may be copyrighted. According to PLOS’s copyright policy, authors who use figures or other material (e.g., graphics, clipart, maps) from another author or copyright holder must demonstrate or obtain permission to publish this material under the Creative Commons Attribution 4.0 International (CC BY 4.0) License used by PLOS journals. Please closely review the details of PLOS’s copyright requirements here: PLOS Licenses and Copyright. If you need to request permissions from a copyright holder, you may use PLOS's Copyright Content Permission form.

Please respond directly to this email or email the journal office and provide any known details concerning your material's license terms and permissions required for reuse, even if you have not yet obtained copyright permissions or are unsure of your material's copyright compatibility.

Potential Copyright Issues:
Figure 2: please (a) provide a direct link to the base layer of the map (i.e., the country or region border shape) and ensure this is also included in the figure legend; and (b) provide a link to the terms of use / license information for the base layer image or shapefile. We cannot publish proprietary or copyrighted maps (e.g. Google Maps, Mapquest) and the terms of use for your map base layer must be compatible with our CC-BY 4.0 license.

Note: if you created the map in a software program like R or ArcGIS, please locate and indicate the source of the basemap shapefile onto which data has been plotted.

If your map was obtained from a copyrighted source please amend the figure so that the base map used is from an openly available source. Alternatively, please provide explicit written permission from the copyright holder granting you the right to publish the material under our CC-BY 4.0 license.

Please note that the following CC BY licenses are compatible with PLOS license: CC BY 4.0, CC BY 2.0 and CC BY 3.0, meanwhile such licenses as CC BY-ND 3.0 and others are not compatible due to additional restrictions.

If you are unsure whether you can use a map or not, please do reach out and we will be able to help you. The following websites are good examples of where you can source open access or public domain maps: 
* U.S. Geological Survey (USGS) - All maps are in the public domain. (http://www.usgs.gov) 
* PlaniGlobe - All maps are published under a Creative Commons license so please cite “PlaniGlobe, http://www.planiglobe.com, CC BY 2.0” in the image credit after the caption. (http://www.planiglobe.com/?lang=enl) 
* Natural Earth - All maps are public domain. (<http://www.naturalearthdata.com/about/terms-of-use/>)

**Reply:**

Thank you. We have recreated the map using Natural Earth public domain data and we have included this information in the map. Please see figure 3.

5. We have noticed that you have uploaded Supporting Information files, but you have not included a list of legends. Please add a full list of legends for your Supporting Information files after the references list.

**Reply:**

This has been added now.

6. If the reviewer comments include a recommendation to cite specific previously published works, please review and evaluate these publications to determine whether they are relevant and should be cited. There is no requirement to cite these works unless the editor has indicated otherwise. 

**N/A**

We would like to thank the reviewers again for the helpful comments and we hope these revisions address their concerns. We appreciate the opportunity to improve our manuscript and remain available for any additional clarifications.

Sincerely,

Paola Cinardo on behalf of all authors
